# Approaching Standardization of Bovine Ovarian Cortex Cryopreservation: Impact of Cryopreservation Protocols and Tissue Size on Preantral Follicle Population

**DOI:** 10.3390/ani16020266

**Published:** 2026-01-15

**Authors:** Paula Romero, Susana Carrocera, Aurora García, Pilar Nieto, Tania Iglesias, Marta Muñoz, Carmen Díez

**Affiliations:** 1 Genética y Reproducción, Servicio Regional de Investigación y Desarrollo Agroalimentario (SERIDA), 33394 Gijón, Spain; paula.romeromunoz@asturias.org (P.R.);; 2 UR CEFIVA, 33011 Oviedo, Spain; 3 Statistical Unit, Scientific-Technical Services, University of Oviedo, 33003 Oviedo, Spain

**Keywords:** ovarian tissue cryopreservation, slow freezing, vitrification, preantral follicles

## Abstract

Cryopreservation of bovine ovarian cortical tissue offers a promising strategy for preserving female fertility and genetic resources, yet outcomes remain variable and influenced by both protocol and tissue size. This study investigated how slow freezing-thawing (SFT) and two vitrification-warming procedures (VW1 and VW2) affect preantral follicle morphology and granulosa cell proliferation in bovine ovarian cortex fragments of two dimensions (1 × 10 × 5 mm and 1 × 10 × 10 mm). Tissue from six cows was processed for histological evaluation and Ki67 immunostaining. Small fragments subjected to SFT showed no significant reduction in the proportion of morphologically normal follicles compared with fresh controls, representing the best overall preservation. In contrast, vitrification decreased morphological integrity, with VW2 performing better than VW1 in both fragment sizes. Small SFT pieces contained more morphologically normal follicles than large ones. Granulosa cell proliferation capacity was largely maintained across cryopreservation protocols, increasing with follicular stage; a size-related difference only appeared on VW2, where small fragments displayed higher Ki67 positivity. These findings underscore the relevance of jointly evaluating cryopreservation protocol and fragment size to optimize bovine ovarian tissue preservation, strengthening the evidence supporting SFT of small fragments as a robust option for safeguarding cortical integrity and improving tissue-based fertility preservation strategies.

## Simple Summary

Cryopreservation of ovarian cortical tissue is a promising tool to preserve the reproductive potential of domestic animals and safeguard genetic resources. This tissue contains a large population of immature preantral follicles that could be used in future reproductive or biotechnological applications. However, the efficiency of current cryopreservation procedures varies widely and is influenced by both the protocol used and the physical characteristics of the tissue fragments. In this study, we compared slow freezing and two vitrification methods using bovine ovarian cortex fragments of two different sizes (small—1 × 10 × 5 mm—and large—1 × 10 × 10 mm—). Our aim was to assess how protocol and tissue dimension affect follicular morphology and granulosa cell proliferation, two indicators of tissue quality after cryopreservation. We found that slow freezing preserved follicle morphology more effectively than vitrification, particularly in smaller tissue fragments. Vitrification performance differed markedly between protocols, with one method substantially reducing morphological integrity. Proliferation activity was generally maintained across treatments, indicating that morphologically normal follicles retain developmental potential after cryopreservation. These results highlight the importance of considering fragment size together with protocol when optimizing bovine ovarian tissue cryopreservation, and support slow freezing of small pieces as an efficient approach for preserving bovine ovarian tissue.

## 1. Introduction

Cryopreservation of ovarian cortex is a valuable strategy for preserving female fertility in both clinical and veterinary fields. In cattle, the reserve of primordial follicles is estimated at approximately 1.5 × 10^5^ at birth [1,2,3]. Throughout postnatal life most of these follicles undergo atresia, and less than 1% reach ovulation, leading to a progressive reduction in the number of available oocytes as the reserve of preantral follicles (primordial, primary, and secondary follicles) is gradually consumed [2]. Since those preantral follicles constitute over 90% of the total ovarian follicle population, this early follicle pool represents the ovarian reserve [3]. The development and optimization of techniques for the long-term preservation of the early follicle pool are therefore essential.

Ovarian cortical tissue cryopreservation (OTC) enables storage of the entire follicular reserve embedded in its native stromal and extracellular matrix environment, offering advantages over isolated oocyte cryopreservation, particularly in species with poor oocyte survival to cryopreservation, or patients for whom ovarian stimulation is not feasible or effective [4,5,6,7,8,9]. Importantly, OTC could potentially allow for the recovery and in vitro culture of a high number of preantral follicles, which after reaching the adequate developmental stage may be matured and used in reproductive biotechnologies [8,10].

In cattle, oocytes show high cryosensitivity due to their high lipid content, low surface-to-volume ratio, and cytoskeletal sensitivity to chilling, which reduces their developmental potential after thawing/warming [11,12,13,14]. These limitations significantly hinder the efficiency of bovine oocyte cryopreservation, reinforcing the rationale for tissue-based preservation approaches. In addition to its application in fertility preservation, bovine OTC plays a key role in the context of animal husbandry and biodiversity conservation. Cryopreservation of ovarian cortex in domestic and wild animals can facilitate the preservation of endangered species and valuable genetic lines, supporting reproductive biotechnologies and long-term genetic banking [15,16,17]. Moreover, bovine ovarian tissue serves as a relevant experimental model for human reproductive medicine due to its morphological and physiological similarities with the human ovary [10,18,19,20,21].

In human medicine, OTC is the only fertility preservation option for prepubertal girls and for patients in whom hormonal stimulation is contraindicated. Although ovarian tissue transplantation after cryopreservation has led to restoration of endocrine function and successful pregnancies, this approach still faces challenges such as ischemic damage post-transplantation and the potential risk of reintroducing malignant cells in oncological patients [10,22,23,24,25]. It should be noted that ovarian tissue transplantation is not a standard practice in domestic or production animals, except within experimental research settings [26].

The two main strategies for OTC are slow freezing (SF) and vitrification (VT). SF involves gradual cooling in the presence of low concentrations of cryoprotectants, which allows for controlled cellular dehydration and extracellular ice formation, reducing the risk of damaging intracellular ice crystals [7,11]. SF is a more established method and has been used in clinical practice, showing relatively consistent results in preserving follicular morphology and even achieving successful tissue transplantation in humans [24,25]. However, there remain some challenges to be solved, as it requires expensive programmable freezing equipment, it is time-consuming (around 3 h), and it may still cause cellular damage due to osmotic stress and residual intracellular ice [10,15,27,28]. VT, on the other hand, is an ultrafast cryopreservation technique that uses high concentrations of cryoprotectants and ultrarapid cooling and warming rates by direct immersion in liquid nitrogen to solidify the tissue into a glass-like state without ice crystal formation [10,11]. This method eliminates the need for expensive equipment and drastically reduces the duration of the procedure (15–45 min, depending on the protocol), making it especially attractive for animal species in field conditions [28,29,30]. VT has been associated with better preservation of stromal and extracellular matrix components compared to SF [10]. However, the high cryoprotectant concentrations used can be cytotoxic and the heterogeneous structure of the ovarian cortex can limit heat and mass transfer during vitrification and warming, resulting in uneven preservation across the tissue [27,30,31].

Despite the widespread use of both techniques, there is currently no standardized protocol for OTC. Studies vary greatly in terms of cryoprotectant types and concentrations, equilibration and exposure times, use of open or closed vitrification systems, and cooling and warming rates [10,25,29,31]. Overall, findings remain divided, some investigations demonstrate better follicular survival following slow freezing [8,10], others find vitrification to be more advantageous [7], and others still observe broadly equivalent results with both techniques [9,25,29,31].

Similarly, there is no consensus on the optimal size of ovarian cortex fragments, which may influence cryoprotectant diffusion, thermal conductivity, and overall tissue preservation [15,23,30]. While many groups standardize slice thickness at 1 mm to balance diffusion and structural integrity, lateral dimensions (length × width) are variably reported and rarely compared directly [7,9,10,29]. A small number of cross-species studies suggest benefits of reduced fragment size for post-thaw morphology ([32], baboon; [33], feline), but bovine data remain sparse and studies often use fragment geometries that are either very small, and therefore difficult to handle, or heterogeneous [34,35], which limits translational applicability and highlights the urgent need for further research aimed at optimizing and standardizing procedures.

Therefore, the objective of this study was to compare the effects of ovarian cortex fragment size and cryopreservation protocol (SF versus two VT approaches) on preantral follicular integrity, through the analysis of follicular morphology and distribution, and the proliferative activity of granulosa cells in bovine ovarian cortex.

## 2. Materials and Methods

All reagents were purchased from Sigma-Aldrich (St. Louis, MO, USA), unless otherwise indicated.

### 2.1. Collection and Processing of Ovarian Tissue

Functional bovine ovaries with visible antral follicles (diameter between 2 and 6 mm) were obtained from six adult Holstein-Friesian cows (>18 months old) at a local slaughterhouse, rinsed in 0.9% (*w*/*v*) NaCl supplemented with penicillin (100 IU/mL) and streptomycin (100 µg/mL), and transported to the laboratory within 2 h at 4 °C in phosphate-buffered saline (PBS).

**Figure 1:**
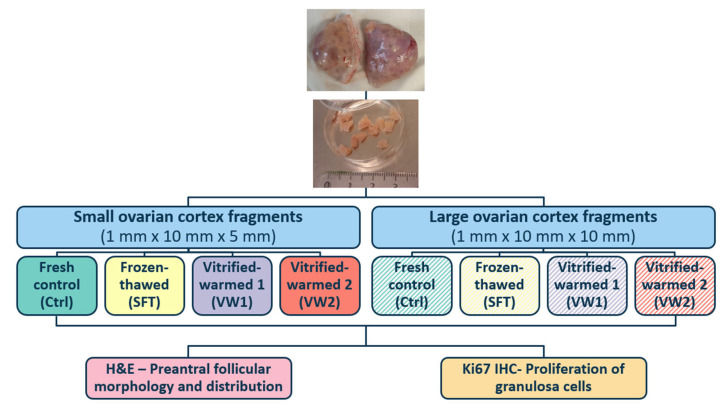
Experimental design.

Once at the laboratory, the ovarian cortex was dissected from the medulla under sterile conditions using a scalpel and dissection scissors and cut into fragments of two sizes (thickness × length × width): large (~1 × 10 × 10 mm) and small (~1 × 10 × 5 mm). Fragments showing morphological damage (hemorrhage, fibrosis, cysts, or discoloration) were excluded, only fragments obtained from regions with similar characteristics were used. Fragments from each ovary and size were randomly allocated to four experimental groups: fresh control (Ctrl), slow frozen-thawed (SFT), vitrified-warmed protocol 1 (VW1) and vitrified-warmed protocol 2 (VW2). A schematic overview of the experimental design is provided in Figure 1.

### 2.2. Slow Freezing and Thawing Protocols

Slow freezing was performed according to Locatelli et al. [9] and Faheem et al. [36], with modifications. Briefly, ovarian cortex fragments were equilibrated in Falcon™ 50 mL conical centrifuge tubes containing 5 mL of PBS with 4 g/L bovine serum albumin (BSA) at room temperature (RT), then 5 mL of freezing solution 1 (FS1: PBS + 4 g/L BSA + 10% DMSO) were added (Corning, Corning, NY, USA). After five minutes of incubation, 5 mL of the solution were retired and another 5 mL of FS1 were added. Fifteen minutes later, 5 mL were retired and 5 mL of freezing solution 2 (FS2: PBS + 4 g/L BSA + 12% DMSO) were added. Finally, after 15 min, fragments were placed in 1.8 mL cryogenic storage vials (Corning^®^ CLS431417, Corning, Corning, NY, USA) containing 1 mL of the FS2 solution. Immediately after, the cryovials were placed in the programmable freezer (Crysalys 9500^®^, Biogenics, Harriman, TN, USA) starting with a 30 min exposure to the cryoprotectant at 4 °C for equilibration. The vials were cooled at a rate of −2 °C /min to −9 °C, held for 2 min, and then, seeded manually. After seeding, samples were cooled to −40 °C at a rate of −0.3 °C/min, and then at −6 °C /min to −70 °C, before plunging into liquid nitrogen (LN_2_).

Thawing was performed at least one week after freezing. For thawing, the cryovials were removed from liquid nitrogen and plunged into a water bath at 42 °C until the content of the vial was thawed and placed in ice. Cryoprotectant was removed by three serial dilutions by removing half of the liquid and gently adding the same volume of PBS with 4 g/L BSA in three steps of 10, 5, and 5 min, respectively.

### 2.3. Vitrification and Warming Protocols

#### 2.3.1. Vitrification-Warming Protocol 1

Tissue pieces were vitrified based on Locatelli et al. [9] and Faheem et al. [36], with modifications. After dissection and preparation, a maximum of four ovarian tissue pieces were placed in Falcon™ 50 mL conical centrifuge tubes, firstly containing 5 mL of holding solution (HS: PBS + 20% fetal calf serum (FCS)). Then, samples were treated at RT as follows:

I. 5 min in 5 mL of vitrification 1—solution 1 (VT1-S1): HS + 5% DMSO + 5% ethylene glycol (EG) + 0.125 M sucrose.
II. 5 min in 5 mL of vitrification 1—solution 2 (VT1-S2): HS + 10% DMSO + 10% EG + 0.25 M sucrose.
III. 2 min in 5 mL of vitrification 1—solution 3: HS + 20% DMSO + 20% EG + 0.5 M sucrose.

At the end of its exposure to cryoprotectant, the tissue was gently picked and inserted using forceps into 1.8 mL plastic cryovials (NUNC 375418, Thermo Fisher Scientific, Waltham, MA, USA) previously pierced with a needle (18 G × 1 ½″), allowing the flow of LN2 into the tube. Finally, samples were plunged directly into liquid nitrogen.

Warming was performed no less than 1 week after vitrification. Cryovials were removed from liquid nitrogen storage and plunged into a water bath at 20 °C. Once the samples were warmed, ovarian tissue fragments were recovered in 35 mm Petri dishes (two fragments per Petri dish) and exposed to 3 mL of VT1-S2 and VT1-S1 (5 min each), and finally washed in 3 mL of HS for 3 min.

#### 2.3.2. Vitrification-Warming Protocol 2

Tissue pieces were vitrified following the protocol Herraiz et al. [7], with minor modifications. A maximum of four ovarian tissue pieces were placed in Falcon™ 50 mL conical centrifuge tubes, firstly containing 5 mL of HS. Then, samples were incubated at RT:

I. 25 min in 5 mL of vitrification 2—solution 1 (VT2-S1): HS + 7.5% DMSO + 7.5% EG + 0.15 M sucrose.
II. 15 min in 5 mL of vitrification 2—solution 2 (VT2-S2): HS + 20% DMSO + 20% EG + 0.5 M sucrose.

At the end of exposure to cryoprotectant, the tissue was gently picked and inserted using forceps into 1.8 mL cryogenic storage vials (NUNC 375418) previously pierced with a needle (18 G × 1 ½″) allowing the flow of LN2 into the tube and plunged directly into liquid nitrogen.

For warming, performed no less than 1 week after vitrification, ovarian tissue fragments were recovered at 37 °C and placed in 35 mm Petri dishes (two fragments per Petri dish) and incubated in HS + 1 M sucrose (3 mL; 1 min; 37 °C), in HS + 0.5 M sucrose (3 mL; 3 min; RT) and in HS (3 mL; 5 min; RT).

### 2.4. Histological Evaluation

Fresh control, frozen-thawed and vitrified-warmed ovarian tissue pieces were placed in 35 mm Petri dishes with fresh PBS (0.1 M PBS, pH 7.4) and were fixed in paraformaldehyde 4% overnight at 4 °C after a previous pre-incubation step of 1 min at 37 °C. Then, fixed samples were dehydrated, embedded in paraffin [37], and sectioned into 5 µm slices for further histological evaluation and immunohistochemistry (IHC).

For the morphological and distribution analyses of preantral follicles, tissue sections were deparaffinized and rehydrated to distilled water before being stained in Mayer’s Hematoxylin for 5 min. Slides were then washed in tap water and counterstained in Yellow Eosin followed by dehydration to 100% ethanol. Slides were cleared twice in Histoclear and mounted in DPX (Eukitt, Bobingen, Germany) before imaging on an Olympus BX51 fitted with an Olympus DP70 digital camera (Olympus, Tokyo, Japan).

Preantral follicles (PFs) within the ovarian tissue pieces were classified based on the morphology of oocytes and granulosa cells [10,30]. Morphologically normal preantral follicles (MNFs) are identified by an intact basal membrane, no pyknotic nuclei, a spherical oocyte and an absence of vacuoles. Atretic degenerating follicles are characterized by pyknotic oocytes or granulosa cells and detachment of the basal membrane [10,30]. MNFs were classified as: primordial follicles, an oocyte surrounded by a single layer of flattened granulosa cells; primary follicles, an oocyte surrounded by a single layer of cuboidal granulosa cells; and secondary follicles, an oocyte surrounded by two or more layers of cuboidal granulosa cells, but without antral cavity.

### 2.5. Immunohistochemistry Analysis for Ki67

Ki67 IHC protocol was performed following established protocol [38] with slight modifications. Briefly, after paraffin removal and rehydration, sections were submitted for antigen retrieval with sodium citrate buffer (10 mM sodium citrate, 0.05% (*v*/*v*) Tween 20, pH 6.0) for 20 min at 95 °C. Subsequently, non-specific binding was blocked in PBS + 5% (*v*/*v*) normal goat serum (NGS). Next, sections were incubated in primary antibody: Rabbit anti-Ki67 (Invitrogen PA5-19462, Invitrogen, Carlsbad, CA, USA) diluted 1 µg/mL in 5% (*v*/*v*) NGS in PBS and incubated overnight at 4 °C. After washing, primary antibody was detected using VECTASTAIN ABC kit following manufacturer instructions (Vector Laboratories, Peterborough, UK). The bound complex was made visible by reaction with 3,3′-diaminobenzidine (DAB). Finally, sections were counterstained in Mayer’s Hematoxylin, dehydrated, cleared and mounted with DPX (Eukitt).

Images were recorded using an Olympus BX51 fitted with an Olympus DP70 digital camera. Ki67-positive follicles (proliferative follicles) were defined by the presence of at least one DAB-stained nucleus among granulosa cells, and follicles with no staining in granulosa cells were classified as Ki67-negative, following established methodology in the literature [10,38]. Negative controls using NGS instead of the primary antibody were included in all the protocols.

### 2.6. Statistical Analysis

Ovaries from six adult cows were used as biological replicates. From each ovary, one cortical fragment of each size (small: 1 × 10 × 5 mm; large: 1 × 10 × 10 mm) was randomly allocated to one of the four experimental groups (Ctrl, SFT, VW1 and VW2), at least 10 slides per group were evaluated, leaving 100 µm between slices. Only follicles with a clearly visible oocyte nucleus were counted and classified to avoid counting the same follicle multiple times, always scored by two independent observers.

A total of 7104 PFs (Ctrl: 1914; SFT: 1773; VW1: 1781; VW2: 1636) were evaluated for follicular morphology and developmental stage analyses. Data were initially tested for normality using the Shapiro–Wilk’s test, and for homoscedasticity with Bartlett’s test. Kruskal–Wallis’s test was used to compare cryopreservation protocols (data violated parametric assumptions), with Dunn’s post hoc test used if significant differences were detected. For comparing the effect of fragment size within each cryopreservation protocol, Student’s t test for independent samples was used (data met parametric assumptions).

Regarding the comparison of the percentages of Ki67-positive follicles, 6071 MNFs (Ctrl: 2037; SFT: 1867; VW1: 668; VW2: 1499) were analyzed. Pearson’s Chi Square test or Fisher’s test were used, depending on the fulfillment of the hypothesis on expected frequencies, performing post hoc test of multiple comparisons with the Bonferroni adjustment.

The significance level used was 0.05 and the statistical analysis was performed with software R, version 4.4.3. Data are presented as mean ± standard deviation for parametric data and as median (25th–75th percentile) for non-parametric data. All statistical computations and validations were performed by the Statistical Consulting Unit of the Scientific-Technical Services at the University of Oviedo.

## 3. Results

### 3.1. Effect of Fragment Size on Preantral Follicular Morphology and Distribution

When pooling data across all cryopreservation protocols, we observed a significant impact of fragment size on preantral follicle morphology and distribution. Specifically, small SFT fragments showed a higher MNFs percentage than large SFT pieces (86.66 ± 10.15 vs. 79.35 ± 13.84, respectively; *p* < 0.001; Table 1).

**Figure 2:**
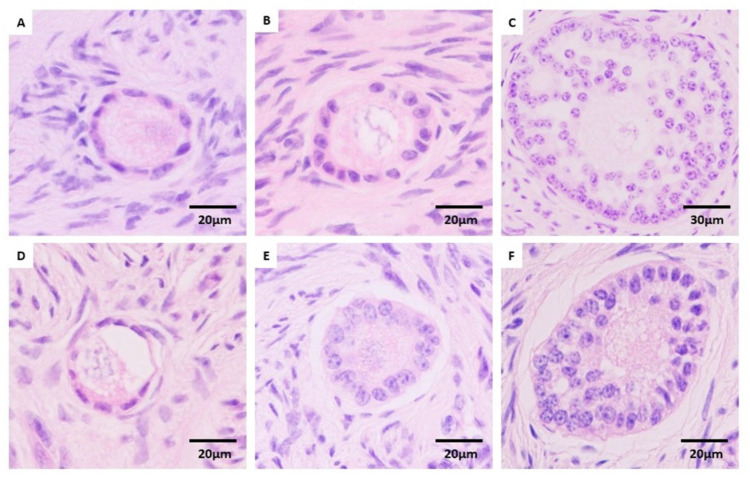
Preantral follicles stained with hematoxylin-eosin. Preantral follicles were categorized into two quality groups: Morphologically normal follicles ((**A**), primordial; (**B**), primary; (**C**), secondary) and atretic follicles ((**D**), primordial; (**E**), primary; (**F**), secondary).

**Figure 3:**
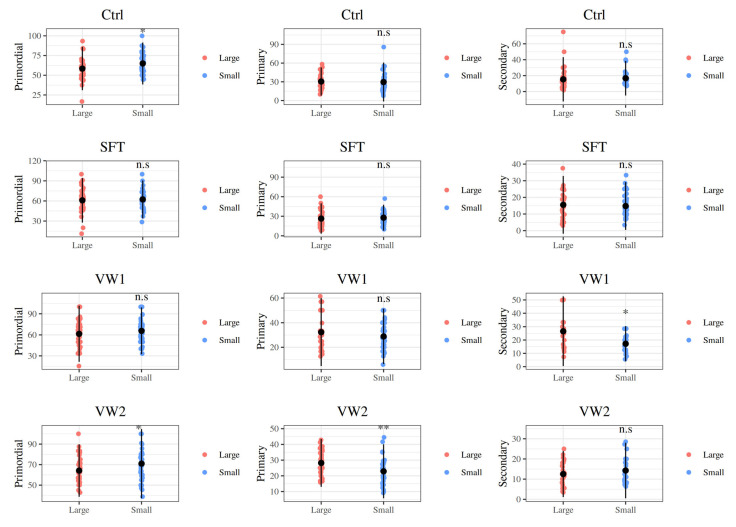
Differences in the distribution of follicular developmental stages (primordial, primary, secondary) between small (1 × 10 × 5 mm) and large (1 × 10 × 10 mm) ovarian cortex fragments following each preservation protocol. Values are expressed as percentage of total counted follicles shown as mean (black dot) and the vertical line is the 95% confidence interval for the mean; * *p* < 0.05, ** *p* < 0.01, n.s not significant.

Regarding the distribution of morphologically normal and atretic follicles at different developmental stages (primordial, primary, and secondary; Figure 2), the effect of fragment size varied depending on the cryopreservation protocol applied (Figure 3). In fresh control tissue (Ctrl), small OTC fragments contained a significantly higher proportion of primordial follicles compared to large fragments (64.93 ± 13.30 vs. 58.45 ± 13.73, respectively; *p* = 0.037). This size-related difference in primordial follicle distribution was also observed in VW2 (70.96 ± 16.88 vs. 64.20 ± 12.77 for small and large, respectively; *p* = 0.045). Conversely, primary follicles were less abundant in small VW2 fragments than in large ones (22.80 ± 8.59 vs. 28.20 ± 7.61, respectively; *p* = 0.007). Regarding secondary follicles, they were less represented in small VW1 fragments than in large ones (17.22 ± 6.61 vs. 26.57 ± 13.07, respectively; *p* = 0.009). No other statistically significant size-related differences were detected across the remaining experimental groups.

### 3.2. Effect of Cryopreservation Procedure on Preantral Follicular Morphology and Distribution

With the aim of isolating the specific effect of the cryopreservation protocol on follicular morphology and distribution without being masked by size-related viability, we delved into the effects of the cryopreservation protocols separately within small and large OTC fragments.

**Figure 4:**
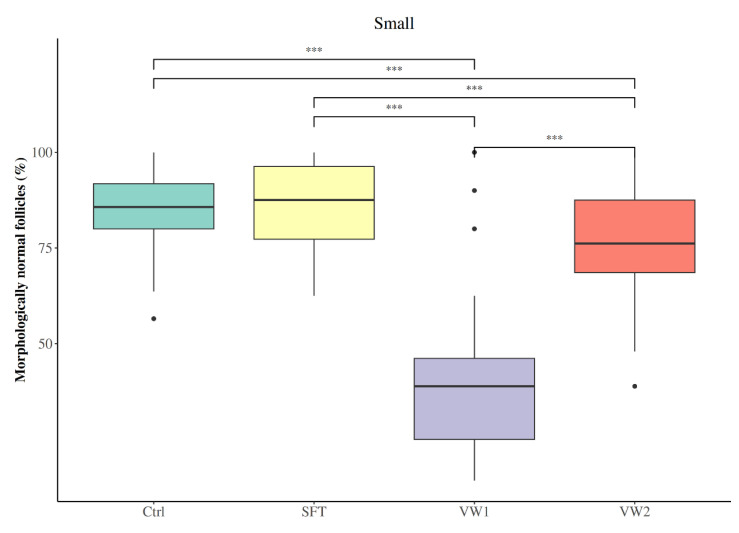
Percentage of morphologically normal follicles (MNFs) in small (1 × 10 × 5 mm) bovine ovarian cortical fragments according to cryopreservation protocol: fresh control (Ctrl), slow freezing-thawing (SFT), vitrification-warming protocol 1 (VW1) and vitrification-warming protocol 2 (VW2). *** *p* < 0.001.

Among small cortical fragments, SFT was the only cryopreservation method that maintained the percentage of morphologically normal follicles (MNFs) at levels statistically indistinguishable from fresh controls (SFT: 87.50 (77.27–96.30); Ctrl: 85.71 (80.00–91.80); *p* = 0.61; Figure 4). In contrast, both vitrification protocols significantly reduced the proportion of MNFs compared to fresh control tissue (VW2: 76.19 (68.57–87.50); VW1: 38.89 (25.00–46.15); *p* < 0.001). SFT preserved a significantly higher MNFs percentage than VW2 (*p* < 0.001) and VW1 (*p* < 0.001). Notably, VW2 significantly improved the percentage of MNFs compared to VW1 (*p* < 0.001). The distribution of follicular developmental stages remained relatively stable across all preservation methods in small fragments (Table 2). Follicular population in all groups of small OTC fragments was dominated by primordial follicles, followed by primary, and lastly secondary follicles. Importantly, no statistically significant differences in the proportion of primordial, primary, or secondary follicles were detected among the four experimental groups.

**Figure 5:**
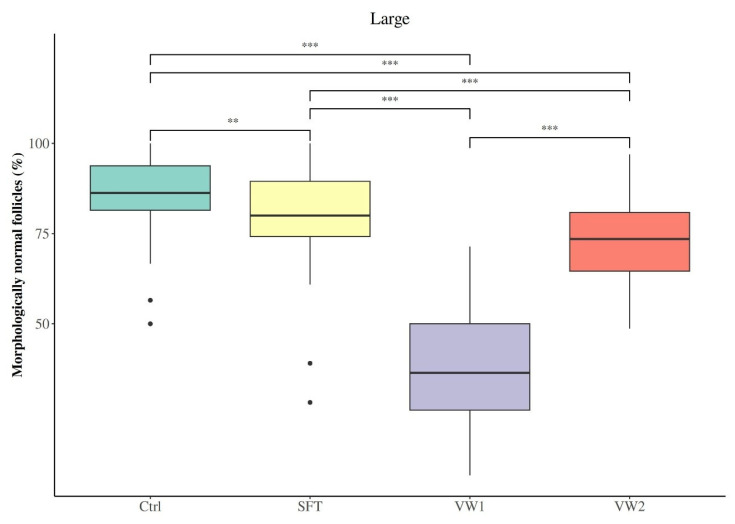
Percentage of morphologically normal follicles (MNFs) in large (1 × 10 × 10 mm) bovine ovarian cortical fragments according to cryopreservation protocol: fresh control (Ctrl), slow freezing-thawing (SFT), vitrification-warming protocol 1 (VW1) and vitrification-warming protocol 2 (VW2). ** *p* < 0.01, *** *p* < 0.001.

Within the large fragments, unlike the pattern observed in small fragments, all three cryopreservation protocols significantly reduced the percentage of morphologically normal follicles compared to the control group (Ctrl: 86.27 (81.48–93.79); SFT: 80.00 (74.19–89.47); VW1: 36.40 (26.09–50.00); VW2: 73.51 (64.60–80.85); Figure 5). Despite this overall reduction, the relative efficacy of protocols followed the same hierarchy observed in small fragments. SFT preserved significantly more MNFs than VW2 and VW1 (*p* < 0.001), while VW2 outperformed VW1 (*p* < 0.001). The morphologically intact follicle pool was predominantly composed of primordial follicles, followed by primary and secondary stages (Table 3). The proportions of primordial and primary follicles did not differ significantly among preservation protocols (*p* > 0.05). However, the proportion of secondary follicles was increased in VW1 relative to Ctrl (*p* = 0.001), SFT (*p* = 0.015) and VW2 (*p* = 0.001).

### 3.3. Effect of Cryopreservation Procedure on Granulosa Cell Proliferation of Preantral Follicles (Ki67 Expression)

**Figure 6:**
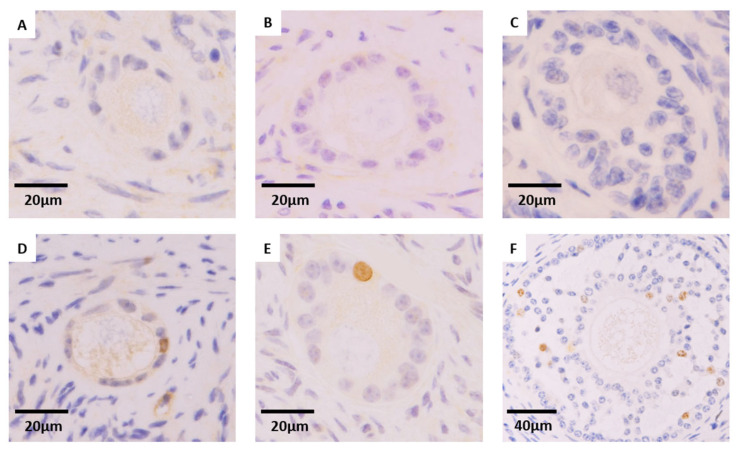
Immunohistochemical staining for Ki67. Preantral follicles were categorized into two quality groups: non-proliferative follicles ((**A**), primordial; (**B**), primary; (**C**), secondary) and proliferative follicles ((**D**), primordial; (**E**), primary; (**F**), secondary).

The proliferative capacity of granulosa cells of morphologically normal follicles at different developmental stages (primordial, primary, and secondary) (Figure 6) was significantly influenced by fragment size (Table 4). Specifically, small VW2 fragments demonstrated a higher percentage of Ki67-positive MNFs than large VW2 pieces (10.79% (15/139) vs. 5.17% (12/232), respectively; *p* = 0.044), although other experimental groups did not yield statistically significant size-related differences.

As in the histological analysis, data for small and large fragments were analyzed and presented separately in order to isolate, and reach a more accurate interpretation, of the protocol-specific effects on follicular proliferation, and to ensure that potential influences related to tissue size and cryopreservation protocol are properly accounted for.

The differentiated analysis of Ki67 expression within the group of small or large samples showed no significant differences between cryopreservation protocols, regardless of follicular developmental stage (Table 5 and Table 6). Both small and large groups showed a progressive significant increase in the Ki67 expression, with follicular development being almost absent in primordial follicles, increasing significantly in primary follicles, and reaching the highest expression within secondary follicles (Table 5 and Table 6).

## 4. Discussion

Our study evaluated how cryopreservation protocol and OCT fragment size influence preantral follicle morphology and distribution, and granulosa cell proliferation in bovine ovarian cortex. By combining a large histological dataset (>13,000 follicles) with H&E evaluation and Ki67 immunostaining, we addressed two complementary dimensions of follicle viability: structural integrity and cellular proliferative potential. Overall, our results indicate that SF of small (1 × 10 × 5 mm) bovine OCT fragments is the most effective protocol among those tested for preserving morphological integrity of preantral follicles, making this approach particularly promising for optimizing ovarian tissue preservation in bovine and related species.

Given the lack of standardization and the contradictory findings regarding the relative efficacy of SF versus VT, rigorous comparative studies are essential to refine OTC protocols. Indeed, the literature presents conflicting conclusions: some studies report superior follicular preservation following SF [8,10], while others demonstrate better outcomes using VT [7] and still others yield similar outcomes with both techniques [9,25,29,31]. These divergent results likely reflect methodological variables that may account for discrepancies such as species-specific differences, variations in cryoprotective agents (CPAs) composition and concentration, equilibration and exposure times, cooling and warming rates, carrier systems for vitrification, fragments dimensions and handling protocols, and evaluation criteria, underscoring the need for direct comparisons under controlled conditions.

In our study, we sought to explore two key variables that may influence cryopreservation outcomes: cryopreservation method and tissue fragment size. In order to assess the efficacy of cryopreservation, we evaluated its effects on follicular morphology and proliferation capacity (Ki67 immunostaining) of granulosa cells within the different preantral follicle developmental stages (primordial, primary, and secondary).

To isolate protocol-specific effects from potential confounding size-related influences, we analyzed and presented data for small and large fragments separately throughout our morphological and proliferative assessments. This stratified analytical approach enabled precise evaluation of each cryopreservation method within each size category, accounting for the known interaction between tissue size and cryopreservation outcomes [32,33,34,35] demonstrated in our study. By avoiding pooled analyses that could mask size-dependent treatment responses, we were able to reveal that protocol efficacy is not uniform across fragment size, a critical finding for standardization efforts.

Follicular damage is one of the main limiting factors in tissue preservation, and morphological assessment remains a reliable and widely accepted method for evaluating follicle viability post-cryopreservation [7,8,9,10,31]. The proliferative capacity of granulosa cells is essential for follicular development, oocyte maturation, and endocrine function. Impaired proliferation can indicate cellular injury or compromised follicular function, even in morphologically intact structures [10,28,38].

Our results demonstrate that the choice of cryopreservation protocol had a significant impact on the percentage of morphologically normal preantral follicles in bovine ovarian cortex. Slow freezing preserved a significantly higher proportion of MNFs, regardless of fragment size, compared to both vitrification protocols. These results align with studies favoring SF in similar large-animal models [8,10]. VW2 consistently outperformed VW1, regardless of fragment size, despite both protocols having identical final CPA concentrations. However, VW2 included longer equilibration times, and a warming protocol beginning at 37 °C with higher sucrose concentrations, likely facilitating faster CPA egress. In small pieces, SFT fragments preserved MNFs at 87.50% (77.27–96.30), statistically indistinguishable from controls 85.71% (80.00–91.80). These findings indicate that while cryopreservation inevitably affects follicular integrity, slow freezing remains more effective in maintaining morphological normality in our experimental setting. These observations agree with previous reports in the literature stating that vitrification outcomes for cortical tissue are highly context-dependent and not uniformly superior to slow freezing [7,8,9,10,25,29,31].

An interesting observation is that the overall distribution of follicle developmental stages (primordial > primary > secondary) remained broadly similar across the four experimental groups, the only exception being an increased proportion of secondary follicles in VW1 large fragments in comparison to Ctrl, SFT and VW2. This pattern likely reflects relative survival bias rather than absolute activation, as VW1 showed a significant loss of MNFs, and such a loss of primordial/primary follicles will increase the relative share of surviving secondaries. Prior reports have observed apparent shifts in class distribution after cryopreservation that were later interpreted as selective follicle loss caused by the vitrification procedure or as massive activation of primordial follicles caused by ischemia–reperfusion after grafting or culture; in this study, neither grafting nor culture were performed, so this alternative is not applicable [10,30,38]. Indeed, variability in follicle stage distribution between individual cortex fragments is well documented, suggesting that intrinsic heterogeneity of follicle density across pieces may contribute to the size-dependent differences we observed in follicular class distribution (Figure 3) [10,39,40,41]. The preservation of stage distribution after SFT and VW2 is encouraging because it suggests that the ovarian reserve (primordial pool) is comparatively protected under those conditions, a key translational objective when OTC is stored for later restoration of fertility or for conservation applications.

The dimensions of ovarian cortex fragments modulate cryoprotectant diffusion, osmotic equilibration, and thermal conductivity. However, the optimal dimensions of ovarian cortical fragments remain poorly defined in the literature, with most studies standardizing thickness but rarely assessing the influence of surface area and lateral dimensions.

In the present study, we compared ovarian cortex fragments of 1 × 10 × 10 mm and 1 × 10 × 5 mm to determine whether reducing the surface area, while maintaining the same thickness, could enhance cryoprotectant penetration and cooling/warming kinetics, ultimately improving tissue preservation. The 1 mm thickness was chosen in accordance with previous studies across species [7,9,10,23,29]. The selection of the two cortical fragment sizes in our study was motivated by the absence of consensus in the literature regarding optimal fragment dimensions for cryopreservation. We selected 10 × 10 mm as it was used in some previously published bovine studies [7,18], and 10 × 5 mm was designed in order to explore whether decreasing tissue area influences post-cryopreservation outcomes, already being a commonly used dimension in human [23]. These dimensions yielded manageable cortical pieces that could be handled and processed reproducibly. Smaller pieces are expected to enhance cryoprotectant penetration and heat transfer, thereby potentially reducing ice crystal formation during slow freezing and improving cooling/warming rates [4,32]. Conversely, overly thin or tiny fragments might compromise tissue handling, increase surface area exposure to CPAs, raising toxicity risk, and disrupt stromal architecture [10,32].

Across species, only a few studies have directly assessed how lateral dimensions of ovarian cortical fragments influence cryopreservation outcomes. In non-human primates, Lu et al. [32] showed that smaller cortical pieces (1 mm × 1–1.5 mm; thick × length and width) yielded significantly higher proportions of morphologically normal primordial follicles after vitrification than fragments of 2 mm in length and width. Similarly in feline ovarian tissue, Gorricho et al. [33] observed improved follicular morphology in the smaller fragments they tested (3 × 3 × 3 mm; thickness × length × width) compared with larger ones (3 × 5 × 3 mm or 3 × 7 × 3 mm; thickness × length × width). Overall, evidence across mammalian species consistently suggests that reducing fragment size enhances cryoprotectant diffusion and thermal homogeneity, improving follicular preservation, although outcomes remain highly protocol dependent.

In cattle, comparable findings have been reported despite the limited number of studies explicitly analyzing tissue dimensions. Ferreira et al. [34] observed that small bovine cortical fragments (3 × 10 × 2 mm; thickness × length × width) exhibited lower percentages of morphologically normal follicles after slow freezing, whereas Dupont et al. [35] found superior follicle morphology in smaller fragments (1 × 3 × 1 mm; thickness × length × width) following cryopreservation and short-term culture. Nevertheless, these bovine studies varied in thickness or overall tissue geometry. Furthermore, considerably smaller and more delicate fragments that are difficult to manipulate, can compromise the tissue (only 66.7% of the primordial follicles in fresh small fragments had a normal morphology [35]) and do not represent the fragment dimensions typically used in applied or translational contexts.

Our results complement and extend the scarce bovine evidence by isolating lateral dimension as the experimental variable while maintaining thickness at 1 mm. We observed that small ovarian cortex fragments generally exhibited superior post-cryopreservation morphological preservation compared to large fragments, particularly following SFT. Critically, the magnitude of the size effect differed between slow freezing and vitrification. Size reduction conferred substantial benefit for SFT, small SFT fragments retained a significantly higher proportion of MNFs than their large counterparts, whereas no significant differences were observed within fresh controls, VW1, or VW2. This improvement can be attributed to fundamental biophysical principles governing ice formation. Smaller tissue volumes enhance thermal conductivity and facilitate more uniform cryoprotectant penetration, also allowing for more controlled dehydration and uniform seeding across the tissue piece. In contrast, the effect of fragment size in vitrification was limited, suggesting that efficacy of vitrification may depend on the balance between the toxicity of high CPA concentrations and vitrification competence. Simply reducing fragment size without simultaneously optimizing CPA protocol and/or temperature may not improve vitrification efficacy. Taken together, the absence of a consistent size effect across all protocols in our study suggests that the interplay between tissue dimensions and specific cryopreservation procedures warrants further investigation, as differences in exposure times, cryoprotectant composition, and cooling rates may modulate the influence of tissue size.

Despite maintaining normal morphology, the functional capacity of cryopreserved morphologically normal follicles may be affected [31]. We assessed Ki67 expression in MNFs as a proxy for granulosa cell proliferative competence, providing a functional complement to morphological evaluation [10,38]. Ki67 is a well-established marker of active cell cycling in granulosa cells; its expression increases as follicles transition from quiescent primordial to activated primary and growing secondary stages [38]. In the present study, proliferative capacity was largely preserved across cryopreservation protocols; no significant differences in Ki67 expression were detected, which is consistent with the literature [10,38]. As expected, Ki67 positivity tracked follicular stage, being almost absent in primordial follicles and highest in secondary ones. This suggests that the subset of follicles that remain morphologically normal after CP retain intrinsic proliferative potential. The only difference detected between fragment sizes was in VW2, where we observed a higher proportion of Ki67-positive follicles in small fragments compared to large fragments. This may reflect intrinsic heterogeneity among the analyzed tissue pieces and/or indicate that small fragments may better preserve proliferative capacity under certain vitrification conditions, possibly due to thermal or diffusion dynamics that remain to be elucidated. Overall, these results suggest that morphologically preserved follicles retain their proliferative potential, underscoring the value of integrating both morphological and functional endpoints when evaluating ovarian tissue cryopreservation.

Literature offers consistent precedent for the aforementioned findings. Amorim et al. [38] found that Ki67 expression can be preserved in surviving follicles after CP, and other studies that combined morphology with cell-cycle or apoptosis markers reported that morphological survivors often retain markers of metabolic and proliferative viability even when overall follicle counts decline [7,9]. However, it should be emphasized that Ki67 positivity is only a proxy for early proliferative ability, and it does not guarantee subsequent oocyte maturation, meiotic competence, or successful growth to ovulation. Prior xenograft and in vitro culture studies [25,30] show that downstream functional assays (steroidogenesis, follicle growth, oocyte maturation) are essential to corroborate the translational relevance of histology and Ki67 findings.

For bovine OTC, our data suggest that small fragments combined with controlled slow freezing are a pragmatic and effective strategy when the objective is to maximize the proportion of morphologically normal follicles that retain proliferative markers. Direct SFT protocol translation for other species requires careful consideration of species-specific anatomical differences; bovine oocyte stands out for its high lipid content compared to other species, such as human [11,12,13,14]. Despite this difference, bovine and human ovarian cortical tissue share remarkable similarities in stromal architecture, follicle distribution, and extracellular matrix composition [10,18,19,20,21]. Noteworthy, both fragment sizes of our study have already been successfully used in human [23]. While our results strongly support slow freezing of small fragments as the optimal protocol for bovine OTC, practical implementation in field settings faces some constraints, such as having approximately three hours of processing time, or the need of a portable programmable freezing equipment, which most of the veterinary teams already use them for in vivo embryo production purposes [10,15,27,28]. Optimized vitrification protocols using the VW2 approach represent a pragmatic compromise when slow freezing is genuinely impossible; VW2 requires only basic laboratory equipment and completes in 45 min. It preserved about 75% of follicle morphology compared to the approximate 85% with SFT, a reduction that may be acceptable in some specific cases.

Follicle density variation among individual ovaries and cortical regions is a well-documented phenomenon [10,39,40,41], which we tried to address through randomization of fragments across treatment groups within each animal but still appeared. Future studies with larger sample sizes (>10 animals or fragments per group) could shed light on whether genetic and physiological variability modulates cryopreservation outcomes. Our vitrification protocols used opened cryovials rather than open carrier systems, which could likely have limited cooling/warming rates. Future research should move beyond morphology and granulosa cell proliferation to incorporate multidimensional assessment of tissue quality and functional competence. Extracellular matrix integrity [7,10], cell viability [7,8,9,10,30,38] and metabolic analyses [7,8,30] could be evaluated, since these elements are increasingly recognized as decisive for graft function and follicle development after transplantation or culture.

## 5. Conclusions

This study provides a systematic evaluation of how cryopreservation protocol and cortical fragment size interact to determine follicle survival in bovine ovarian tissue. By analyzing over 13,000 preantral follicles and combining morphology with granulosa cell proliferation, we demonstrated that tissue size is a determinant factor of cryosurvival. Particularly, slow freezing of small cortical fragments (1 × 10 × 5 mm) preserved a proportion of morphologically normal follicles similar to fresh tissue and significantly higher than large fragments subjected to the same protocol, underscoring the critical role of fragment dimensions in optimizing outcomes. In contrast, vitrification performance was strongly protocol-dependent, with VW2 offering partial improvement over VW1 but not matching slow freezing in either fragment size. Importantly, Ki67 expression was preserved across all cryopreservation conditions, indicating that morphologically normal follicles retain proliferative activity and suggesting that cryopreservation does not compromise early granulosa cell function.

Altogether, these findings contribute new, species-specific insights into how fragment size and cryopreservation strategy interact in bovine ovarian cortex, a model of high translational value for both reproductive biotechnology and human fertility preservation. Small cortical fragments combined with slow freezing as a reliable approach for maintaining structural and functional follicle integrity under current technological constraints.

Future research should move beyond morphology and proliferation to assess cellular viability, extracellular matrix integrity, and stromal support, since these elements are increasingly recognized as decisive for graft function and follicle development after transplantation or culture. Integration of histological, molecular, and functional endpoints will be essential to further refine protocols and to translate bovine and other large-animal models into clinically robust strategies for ovarian tissue preservation.

## Figures and Tables

**Table 1:** Comparison of the percentage of morphologically normal follicles (MNFs) between small (1 × 10 × 5 mm) and large (1 × 10 × 10 mm) bovine ovarian cortical fragments for each cryopreservation protocol.

	Follicles with Normal Morphology (%)			
Group	Ctrl	SFT	VW1	VW2
Small	85.09 ± 10.98	86.66 ± 10.15 ^a^	39.28 ± 17.93	76.19 ± 13.29
Large	85.50 ± 10.67	79.35 ± 13.84 ^b^	38.27 ± 15.80	72.72 ± 11.59

Ctrl: fresh control; SFT: slow freezing-thawing protocol; VW1: vitrification-warming protocol 1; VW2: vitrification-warming protocol 2. Data are presented as mean ± standard deviation. Different lowercase letters indicate significant differences between fragment size within the same protocol (*p* < 0.001).

**Table 2:** Distribution of follicular developmental stages (primordial, primary, secondary) in small ovarian cortex fragments following each preservation protocol.

Small	Preantral Follicle Developmental Stage (%)		
Group (N of MNFs)	Primordial	Primary	Secondary
Ctrl (635)	65.37 (55.41–72.65)	26.09 (20.00–37.04)	13.39 (10.00–19.17)
SFT (618)	63.07 (51.04–70.57)	26.67 (22.25–33.33)	13.33 (10.00–18.54)
VW1 (362)	66.67 (55.56–72.73)	27.78 (21.11–35.68)	16.67 (12.50–21.11)
VW2 (475)	69.34 (59.12–80.00)	21.11 (17.80–27.60)	12.13 (8.33–19.55)

Values are expressed as percentage of total counted MNFs and presented as median (P25–P75). Fresh control (Ctrl), slow freezing-thawing (SFT), vitrification-warming protocol 1 (VW1) and vitrification-warming protocol 2 (VW2).

**Table 3:** Distribution of follicular developmental stages (primordial, primary, secondary) in large ovarian cortex fragments following each preservation protocol.

Large	Preantral Follicle Developmental Stage (%)		
Group (N of MNFs)	Primordial	Primary	Secondary
Ctrl (1018)	59.09 (50.00–66.67)	30.36 (25.00–34.62)	13.12 (7.57–20.71) ^a^
SFT (793)	62.20 (50.32–69.81)	25.00 (19.20–31.44)	15.79 (7.02–17.27) ^a^
VW1 (248)	62.50 (50.00–75.00)	31.67 (20.56–38.33)	25.00 (15.48–33.33) ^b^
VW2 (716)	62.50 (54.80–71.43)	28.08 (22.22–33.65)	12.50 (8.33–16.53) ^a^

Values are expressed as percentage of total counted MNFs and presented as median (P25–P75). Different lowercase letters indicate significant differences between protocols within each fragment size (VW1 vs. Ctrl (*p* = 0.001); VW1 vs. SFT (*p* = 0.015); VW1 vs. VW2 (*p* = 0.001)).

**Table 4:** Percentage of Ki67-positive follicles in primary and secondary follicles from small (1 × 10 × 5 mm) and large (1 × 10 × 10 mm) bovine ovarian cortical fragments after cryopreservation.

Ki67-Positive Follicles (%)				
Group	Primary		Secondary	
	Small	Large	Small	Large
Ctrl	5.07% (11/217)	5.20% (17/327)	46.67% (42/90)	38.60% (44/114)
SFT	6.03% (14/232)	5.07% (11/217)	48.51% (49/101)	50.94% (54/106)
VW1	3.06% (3/98)	3.03% (2/66)	54.55% (36/66)	50% (15/30)
VW2	10.79% (15/139) ^a^	5.17% (12/232) ^b^	60.32% (38/63)	52.69% (49/93)

Fresh control (Ctrl), slow freezing-thawing (SFT), vitrification-warming protocol 1 (VW1), and vitrification-warming protocol 2 (VW2). ^a,b^ Values with different superscripts differ significantly (*p* = 0.044).

**Table 5:** Granulosa cell proliferation indicated by the total percentage of Ki67-positive follicles in primordial, primary, and secondary follicles from small (1 × 10 × 5 mm) bovine ovarian cortical fragments for each cryopreservation protocol.

Small	Ki67-Positive Follicles (%)			
Group	Primordial	Primary	Secondary	
Ctrl	0% (0/515) ^a^	5.07% (11/217) ^b^	46.67% (42/90) ^c^	*p* < 0.001
SFT	0% (0/556) ^a^	6.03% (14/232) ^b^	48.51% (49/101) ^c^	*p* < 0.001
VW1	0% (0/250) ^a^	3.06% (3/98) ^b^	54.55% (36/66) ^c^	*p* < 0.001
VW2	0% (0/419) ^a^	10.79% (15/139) ^b^	60.32% (38/63) ^c^	*p* < 0.001

Fresh control (Ctrl), slow freezing-thawing (SFT), vitrification-warming protocol 1 (VW1), and vitrification-warming protocol 2 (VW2). ^a,b,c^ Values with different superscripts differ significantly between columns.

**Table 6:** Granulosa cell proliferation indicated by the total percentage of Ki67-positive follicles in primordial, primary, and secondary follicles from large (1 × 10 × 10 mm) bovine ovarian cortical fragments for each cryopreservation protocol.

Large	Ki67-Positive Follicles (%)			
Group	Primordial	Primary	Secondary	
Ctrl	0.26% (2/774) ^a^	5.20% (17/327) ^b^	38.60% (44/114) ^c^	*p* < 0.001
SFT	0% (0/655) ^a^	5.07% (11/217) ^b^	50.94% (54/106) ^c^	*p* < 0.001
VW1	0% (0/158) ^a^	3.03% (2/66) ^a^	50% (15/30) ^b^	*p* < 0.001
VW2	0% (0/553) ^a^	5.17% (12/232) ^b^	52.69% (49/93) ^c^	*p* < 0.001

Fresh control (Ctrl), slow freezing-thawing (SFT), vitrification-warming protocol 1 (VW1) and vitrification-warming protocol 2 (VW2). ^a,b,c^ Values with different superscripts differ significantly between columns.

## Data Availability

Data set will be openly available in Zenodo.
